# Increased pollen source area does not always enhance the risk of pollen dispersal and gene flow in *Oryza sativa* L

**DOI:** 10.1038/s41598-020-63119-z

**Published:** 2020-04-09

**Authors:** Ning Hu, Xiaodong Jiang, Qianhua Yuan, Wuge Liu, Kemin Yao, Yan Long, Xinwu Pei

**Affiliations:** 1 Yale-NUIST Center on Atmospheric Environment, International Joint Laboratory on Climate and Environment Change, Nanjing University of Information Science & Technology, Nanjing, 210044 China; 2 Jiangsu Key Laboratory of Agriculture Meteorology, School of Applied Meteorology, Nanjing University of Information Science and Technology, Nanjing, 210044 China; 3 College of Tropical Agriculture, Hainan University, Haikou, 570228 China; 4 Rice Research Institute, Guangdong Academy of Agricultural Sciences, Guangzhou, 510640 China; 5 Biotechnology Research Institute, Chinese Academy of Agricultural Sciences, Beijing, 100081 China

**Keywords:** Biotechnology, Plant sciences

## Abstract

Pollen dispersal is one of the main ways of gene flow. In the past years, rice pollen dispersal and gene flow have been well studies. However, there is much dispute whether the risk of pollen dispersal and gene flow continuously increases with the source area. A Lagrangian stochastic model was used to simulate the pollen depositions at different distances from different pollen source areas. The field experiments showed a good fit in the pollen depositions. The larger the source area, the more the pollen grains were deposited at each distance, with the pollen dispersal distance increasing accordingly. However, this effect gradually leveled off as the source area increased. In the large-area of pollen source, we found a significantly higher saturation point for the amount of pollen deposition. Once the source area exceeded 1000 × 1000 m^2^, the pollen deposition no longer increased, even if the source area continued to increase, indicating the “critical source area” of rice pollen dispersal. However, a 100 × 100 m^2^ critical source area for conventional rice and hybrid rice was sufficient, while the critical source area for the sterile line was about 230 × 230 m^2^.

## Introduction

Gene flow is known as an integrated process in which pollen of a particular donor diffuses to the stigma of a recipient plant by way of wind, insects, or wind and insects, and then fertilizes to produce seeds. In the natural world, gene flow often occurs between cultivars and their wild relatives. Gene flow is a naturally occurring universal phenomenon that is the driving force behind evolution.

There are a number of reasons that the gene flow in rice has long been a concern. First, wild rice is abundant in Southern China and there is concern over whether transgene flow affects the biodiversity of common wild rice. Second, China is a large hybrid-rice-producing country, and gene flow to sterile lines can affect seed purity and inflict economic loss. Finally, insect-resistant transgenic rice lines have obtained biosafety certificates in China, while GM rice with other traits has also entered field testing stages^1^, and their potential risks to the environment and food safety are widely concerned.

The risk of gene flow is an important part of transgenic rice environmental safety assessment, it is very important to master the rule of gene flow in setting reasonable isolation distances between transgenic and no transgenic rice. In the past 20 years, GM rice or rice with certain morphological markers have been used at home and abroad to study the law of gene flow. The gene flow frequency is identified whether the individual plants are produced by gene flow. The relationship between gene flow frequency and distance, as well as the maximum gene flow frequency and gene flow distance, are confirmed^2–8^. The influence of wind speed, direction and rice species on the gene flow frequency have also been clarified^9–14^.

These researches above provided a theoretical basis for setting the isolation distance. However, a common issue in these experiments is that the pollen source areas are only 0.3–1040 m^2^ ^3,6,8,9,11,14–17^. Previous studies showed that the larger the source area, the higher the pollen concentration at each distance, and the greater the possibility that a pollen grain falls on the stigma of cultivated rice or common wild rice, representing a much higher risk of gene flow^15,16^. Therefore, we believe that the results of these small field experiment somewhat underestimate the risk of gene flow in rice and that the data do not accurately represent the gene flow frequency in large-scale commercial production of GM rice.

It is very necessary to analyze the effects of pollen sources areas on rice pollen dispersal and gene flow. Isolation measures must be taken to prevent interaction between the treatments and the surrounding rice. However, distance isolation will result in the field area that is too large to operate practically and time isolation will result in inconsistent environmental conditions between the treatments. Therefore, a combination of a field experiment with a mathematical model is a more effective way to analyze the effects from different pollen sources area.

Gene flow in *Oryza sativa* L. is a synthesis of physical and biological processes. It is a physical process during the pollen grains shed from the anthers of donor, diffused in the atmosphere and then deposited in the canopy or on the ground, while the donor pollen fertilized on recipient stigma and then produced seeds is a biological process. Therefore, the model is made up of these two parts^15,18,19^. Compared with maize, there is relatively little research on rice pollen dispersal and gene flow. Globally, only two research groups have established gene flow model for rice: one is a semi-mechanism model by Rong *et al*.^15^ and Wang *et al*.^18^. The other is based on the Gaussian plume model by Yao *et al*.^19^. However, they are both a negative exponential function, which show a limitation in the complex environment. In addition, the water content and crossing trajectory effect (CTE) of pollen grains failed to account in the model. The water content of pollen grains for rice directly affects its dispersal in the air through the settling speed. Due to dehydration, the longer pollen is exposed to air, the lower the water content is. Aylor^20^ reported that the settling speed of maize pollen after complete dehydration is 34% lower than that of fresh pollen. Rice pollen also has a large particle size and may not completely follow a turbulent motion, forming a “crossing trajectory effect (CTE)”. If CTE is not considered, the pollen deposition is underestimated near the pollen source and overestimated far away from the source^21^. Our study, based on the Lagrangian stochastic (LS) model, establishes a rice pollen dispersal model, in addition, the water content and the CTE of the pollen grains for rice are considered. This model is used to compare the pollen distribution under different pollen source areas, and analyze the effect of source area on the pollen dispersal, then identify whether there is a “critical source”.

## Results

### Model validation

**Figure 1:**
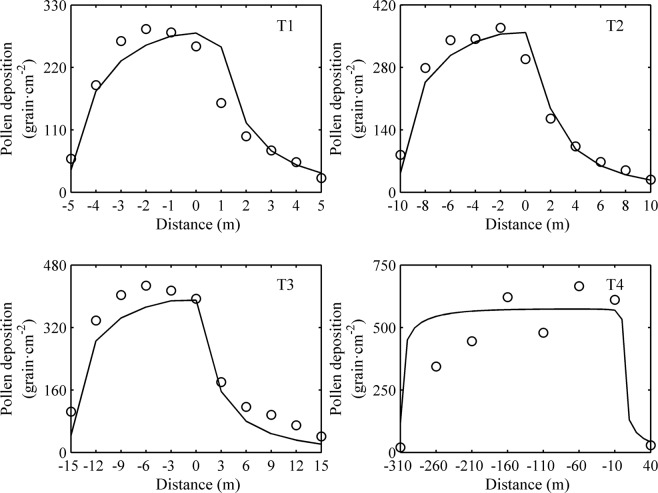
Validation of the pollen dispersal model. Open circles are the measured values of pollen deposition in the field experiment; the solid line is the simulated values calculated by the pollen dispersal model.

The pollen depositions at different locations in the treatments of T1-T4 were simulated by the rice pollen dispersal model above, which were compared with those measured in the field experiments. The simulated value had the same variation characteristics with the measured value (Fig. 1). The fitting equation between them was *y* = 0.9487*x* (n = 41; P ≤ 0.01), and the root mean square error RMSE = 99.9 grain·cm^−2^, with the correlation coefficient r = 0.8456.

Taking T1-T3 as an example, we contrasted the results of before and after accounting for the CTE and water content of pollen grain in our model (Table 1). After correcting the CTE, the RMSE from the simulated value only reduced by 6.8%. This was because the diameter of a single rice pollen grain was only 42 μm and this effect could be negligible compared to a heavier pollen grain such as maize pollen^22^. After introducing the pollen water content into our model, the RMSE of the simulated value increased slightly instead. This was likely because the pollen dispersal distance in this modelling was very short (Table 1) and there was a small change in pollen water content. However, when the dispersal distance reached 10 m or more, the gap between the simulated and the measured value was gradually enlarging with the dispersal distance. Therefore, the pollen water content needed to be treated separately in different conditions. For the sterile line, the distances at which the gene flow frequency was equal to or lower than the threshold value 1% or 0.1% reached from tens to hundreds of meters^12^, and thus the model should consider these changes in pollen water content. For the hybrid and conventional rice, 0.1% threshold distances were generally within 5 m in southern rice region of China^19^, and the pollen water content could be ignored in the model.

**Table 1:** Crossing trajectory effect (CTE) and water content of pollen grains are included and not included in the rice pollen dispersal model.

Case	Fitting equation	r	RMSE(grain·cm^−2^)
CTE and water content not included	y = 0.927 × −4.983, p < 0.01	0.9682	38.0
Only CTE included	y = 0.955 × −5.153, p < 0.01	0.9687	35.4
Only water content included	y = 0.927 × −5.130, p < 0.01	0.9681	38.2
CTE and water content all included	y = 0.959 × −5.604, p < 0.01	0.9683	35.6

Note: r is the correlation coefficient, RMSE is the root mean square error.

### Effect of pollen source area on pollen deposition

**Figure 2:**
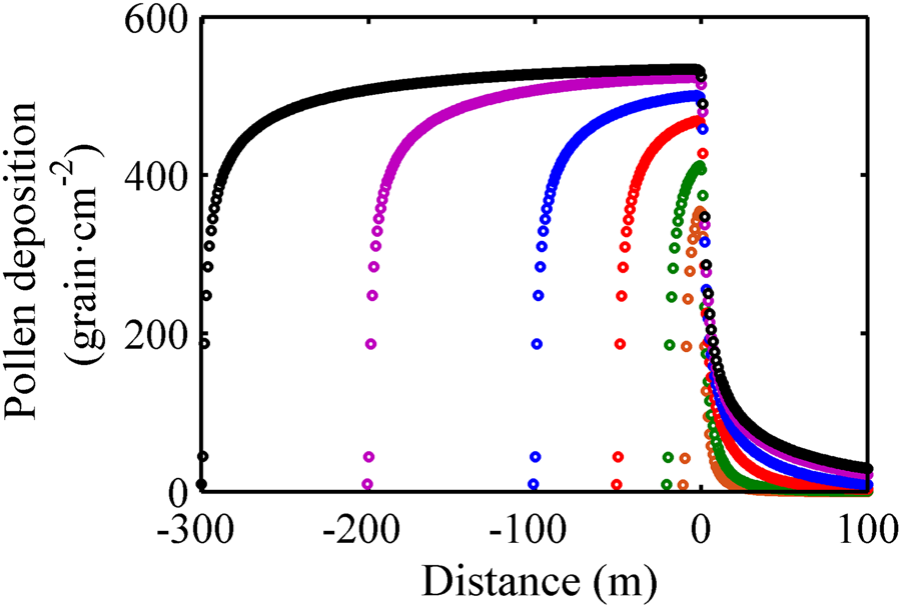
Simulation results of pollen depositions at different distances for the cases with different source area. Different colors indicate different source areas. Brown represents case 7, its source area is 10 × 10 m^2^; green represents case 8, its area is 20 × 20 m^2^; red is case 9, its area is 50×50 m^2^; blue is case 10, its area is 100 × 100 m^2^; purple is case 11, its area is 200 × 200 m^2^; black is case 12, its area is 300 × 300 m^2^.

The different source area of 10 × 10 m^2^, 20 × 20 m^2^, 50 × 50 m^2^, 100 × 100 m^2^, 200 × 200 m^2^ and 300 × 300 m^2^ under the same meteorological conditions were used as the model input to make the contrast of the pollen deposition at different locations. The results were shown in Fig. 2.

The spatial characteristics of pollen depositions was similar across the different cases with different areas of pollen sources. Each rice plant could be regarded as a point source. For each point source, the pattern of pollen dispersal followed a negative exponential distribution, in other words, most of pollens grains deposited within a close distance from this point source. So, the closer it was to the point source, the more the pollen deposition. The pollen deposition measured in the experiment was added by the pollen grains from each point sources upwind. It was noted that the pollen deposition was not simply multiplied by the size of pollen source. Owing to the different location for each pollen source, the pollen grains deposited on a given observation site from a single pollen source was not same as those from another pollen source. It’s for these reasons above, along the prevailing wind direction, firstly, there was a rapid rise of about 15 m in pollen deposition within the pollen source. And then, the pollen depositions were slowly rising until it reached the highest value within the source area less than 100 × 100 m^2^; for larger than 100 × 100 m^2^ of source area, the pollen depositions increased only in the first 100 m, and then almost stop until to the edge of pollen source. This implied that most of the pollen grains could not travel more than 100 m in the air and there was a saturated field which the pollen depositions were almost no longer increasing or decreasing.

However, a significantly difference lied in the order of pollen deposition. We compared the results from different cases and found that the farther away from the pollen source, the more the pollen deposition increased. Compared with the source area with 10 × 10 m^2^, the pollen depositions at the edge of source area increased by 15.5%, 31.0%, 39.7%, 46.1% and 48.9% for the source area of 20 × 20 m^2^, 50 × 50 m^2^, 100 × 100 m^2^, 200 × 200 m^2^ and 300 × 300 m^2^. At the distance of 100 m away from the pollen source, the results were similar, and the pollen deposition increased by 2.2–148.0 times.

In particular, the pollen depositions did not increase in proportion to the source area. For example, the source areas with 20 × 20 m^2^, 50 × 50 m^2^, 100 × 100 m^2^, 200 × 200 m^2^ and 300 × 300 m^2^ increased by 3–899 times more than that of 10 × 10 m^2^, but the maximum pollen depositions increased by only 16.4–50.9%. Obviously, this increase gradually leveled off as the source area increased.

### The effective source and ineffective source

The pollen grains that escaped above the rice canopy could be transported downward the wind direction. Some of these pollen grains would fall within the source area, which would not result in the gene flow from the pollen donor to other recipients, so called “ineffective source”. Others could be transported to a greater distance, and if they dispersed outside of the pollen source and fallen on the stigma of other cultivated rice or its wild relatives, the gene flow phenomenon might occur, so called “effective source”.

Here, we accumulated the total pollen grains fallen within and outside of the source area to obtain the ineffective source and effective source, respectively. In Table 2, RE was used to indicate the ratio of effective source, and DP_50–99%_ represents the pollen dispersal distance at a given probability of 50–99%, in other words, 50%-90% pollen grains in the effective source would be deposited within the range from the edge of source area to the distance of DP_50–99%_.

**Table 2:** The ratio of effective source (RE) and the pollen dispersal distance at a given probability of 50–99% (DP_50–99%_) for different cases.

Source area	Wind speed	RE (%)	DP (m)				
			50%	75%	90%	95%	99%
10 × 10 m^2^	0.5 u	51.3	7	29	91	157	320
	1.0 u	53.4	10	43	148	270	577
	1.5 u	53.7	11	52	192	361	800
20 × 20 m^2^	0.5 u	39.0	10	40	112	184	352
	1.0 u	41.8	14	60	186	320	635
	1.5 u	42.6	16	73	242	430	881
50 × 50 m^2^	0.5 u	25.2	16	58	146	225	399
	1.0 u	28.6	24	91	246	394	725
	1.5 u	29.6	28	114	326	535	999
100 × 100 m^2^	0.5 u	17.1	22	73	170	253	433
	1.0 u	20.4	33	120	298	451	791
	1.5 u	21.6	41	155	401	621	1093
200 × 200 m^2^	0.5 u	10.9	27	86	190	274	457
	1.0 u	13.9	45	151	345	503	843
	1.5 u	15.2	57	199	473	697	1172
300 × 300 m^2^	0.5 u	8.0	30	92	197	282	465
	1.0 u	10.7	52	166	366	527	865
	1.5 u	12.0	68	225	508	734	1204

Note: Effective source represents the pollen grains dispersed and deposited outside of the source area, and ineffective source is the opposite of effective source, and it represents the pollen grains deposited within the source area. RE represents the ratio of effective source. DP_50–99%_ represents the pollen dispersal distance at a given probability of 50–99%, in other words, 50%-90% pollen grains in the effective source would be deposited within the range from the edge of pollen source to the distance of DP_50–99%_.

Table 2 showed that the RE had an apparent difference among the cases with different areas of pollen source. First, the larger the source area, the smaller the RE, i.e., a smaller proportion of pollen grains deposited outside of the pollen source. Second, the larger the source area, the larger the pollen dispersal distance, i.e. a wider range for the locations of pollen grains deposited. However, this trend also gradually leveled off. For example, the source area with 20 × 20 m^2^ increased by 3 times than that of 10 × 10 m^2^, and the pollen dispersal distance only increased by 26%, while the source area with 50 × 50 m^2^ increased by 24 times and the pollen dispersal distance increased by 66%, and so on.

Wind was the driving force to carry the pollen grains and this was the most important meteorological factor to affect the pollen dispersal. Thus, we compared the results under different wind speed conditions. Both RE and DP increased with increasing wind speed. With the source area of 10 × 10 m^2^ as an example, when the wind speed increased from 0.5 × u to 1 × u and 1.5 × u, RE increased by 4% and 5%, respectively, and DP_90%_ increased by 63% and 111%, respectively. When the source area reached to 300 × 300 m^2^, RE increased by 21% and 23%, respectively, and DP_90%_ increased by 86% and 158%, respectively. This trend was even more obvious in the larger-size of pollen sources.

### Critical source area of rice pollen dispersal

**Figure 3:**
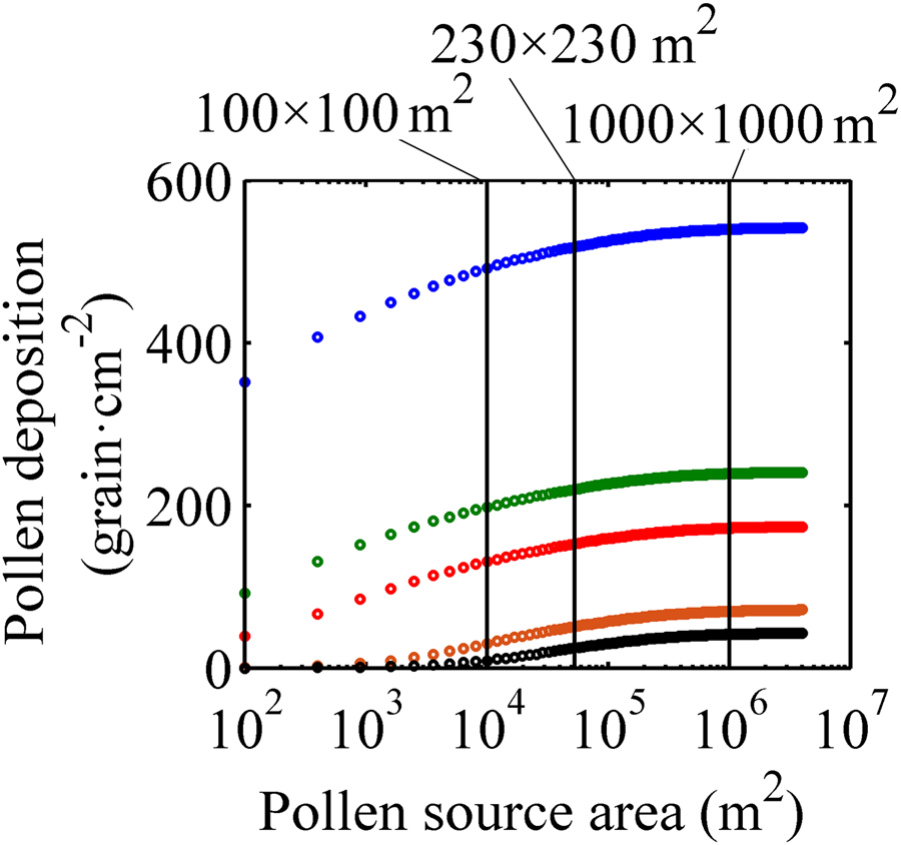
Relationship between the pollen deposition and the source area. Different colors indicate different locations of pollen deposition. Blue, green, red, brown and black indicate 0m, 5 m, 10 m, 50 m, and 100 m distance downwind from the source area, respectively.

We used a group of cases with different areas of pollen source as the model input to calculate the pollen depositions at different locations and to obtain the relationship between the source area and the pollen deposition. The results, shown in Fig. 3, indicate that at the same distance from the pollen source, the pollen deposition increased as the source area was increasing. However, when the pollen source was larger than 1000 × 1000 m^2^, the pollen deposition was almost no longer increased. Therefore, 1000 × 1000 m^2^ could be considered as the “critical source area” for the rice pollen dispersal in our study.

When the source area increased to 100 × 100 m^2^, a turning point appeared, where the data showed a significant slowdown in the increase of pollen deposition. Compared with the source area of 1000 × 1000 m^2^, the pollen deposition at the edge of the source area differed by less than 50 grain·cm^−2^. Therefore, the number of pollen grains deposited on a stigma with 2.5 mm length and 1.0 mm width differed by less than one pollen. Although each stigma could be only eventually fertilized with a single pollen, the more the pollen grains that fall onto the stigma, the greater the probability of fertilization. When the recipient is a conventional rice, which has its own of pollen grains, this single pollen from the donor may find it difficult to compete with the pollen grains from the recipient itself, and thus there was no difference in the fertilization between the donor of 100 × 100 m^2^ and 1000 × 1000 m^2^. It suggests that the “critical source area” of 100 × 100 m^2^ is sufficient for the gene flow to cultivated rice.

The gene flow to sterile lines was another case as these lines have no fertile pollen of their own, but served as an “amplifier” of gene flow, and the gene flow frequency to a sterile line was 1–2 orders of magnitude higher than that to the conventional varieties^23^. When the recipient was a sterile line and there was no competition with the pollen from the donor, the gene flow frequency was theoretically 100%. Gene flow frequency depended on the ratio of donor pollen in the mixed pollen. The larger the competition pollen source area, the greater the area effect of the pollen source. As the competition pollen area increased, the “critical source area” of gene flow to the sterile line would also increase. During practical production, rice is often grown in a large scale, and the competitive pollen was saturated in the pollen deposition. In this case, when the pollen source area reached to 230 × 230 m^2^, the increase in the ratio of donor pollen to the mixed pollen was reduced to less than 1%. Therefore, the area effect of pollen source on the gene flow to sterile line was very small, and 230 × 230 m^2^ could serve as the “critical source area” for sterile lines.

## Discussion

The law of gene flow frequency was the basis for setting the isolation distance during the coexistence of transgenic and non-transgenic rice. A lot of studies on gene flow were conducted in recent years to better understand the law and its consequences.

Maize was one of the most important genetically modified (GM) crops to be commercialized, and the studies on the maize gene flow provided us as a reference. After the industrialization of GM maize, researchers in Europe collected samples from non-GM maize at different distances surrounding the large-scale transgenic maize planting areas to study the coexistence of GM maize and non-GM maize. The distances at which the gene flow frequency was equal to or lower than the threshold value 1% and 0.1% were generally not more than 30 m and 80 m^24,25^, respectively. Comparing the data from the field experiments on maize gene flow frequency^26–30^; indicated that there was no significant increase in the threshold distance. Therefore, the risk of gene flow in crops did not increase indefinitely as the increase of source area.

In this study, we used the rice pollen dispersal model to explore the relationship between the source area and the pollen deposition. We found that the pollen deposition and the pollen dispersal distance did not rise in proportion with the larger pollen source area, while RE gradually decreased. It should be pointed out that the effect leveled off with larger areas of pollen source. This phenomenon was also proved in field experiments. We thus speculate that there was a “critical source area” in rice pollen dispersal. This study further showed that the critical source area was 1000 × 1000 m^2^. If the actual source area exceeded this critical value, the pollen deposition would be almost unchanged. However, the maximum pollen source area was only 72 m in Song *et al*.^16^, which was much smaller than the critical area. The observed differences are likely because material with lower outcrossing rate was used as the pollen recipient in Dong *et al*.^31^, which reduced the area effect of pollen source. The current study showed that when hybrid and conventional varieties were used as the recipients, the effect was reduced as the pollen source went beyond the critical value of 100 × 100 m^2^. Rong *et al*.^15^ also reported the similar results that the effect was not obvious for length of pollen source larger than 100 m. In this study, due to the amplification of sterile line as the recipient, the area effect of pollen source was enhanced. However, this effect was very restricted for the larger pollen sources greater than 230 × 230 m^2^.

The pollen source areas in previous field experiments with rice were: 0.3–72 m^2^ ^11^, 0.6–10 m^2^ ^16^, 3 m^2^ ^8^, 7 m^2^ ^3^, 10 m^2^ ^14^, 13–20 m^2^ ^6^, 25–100 m^2^ ^9^, 800 m^2^ ^15^, 1040 m^2^ ^17^, which were much smaller than the critical source area. When the recipients were adjacent to the donors, only one row of donor could provide enough pollen grains to the recipient. Thus, an increase of source area had very little effect on the maximum gene flow frequency. As the distance increased, pollen deposition showed a negative exponential decay, and the pollen grains were no longer saturated for fertilization. The larger the pollen source area, the more pollen grains from the donor were deposited onto the stigma of the recipient, increasing risk of gene flow accordingly. Therefore, the farther away from the pollen source, the more significant the area effect of pollen source. This showed that a small-size pollen source significantly underestimated the gene flow distance and this underestimation was more severe at the lower threshold used. These conclusions could optimize the design of field experiments and help to assess the risk of the rice transgenic flow as well as solve the seed purity problems encountered in the hybrid rice production.

## Methods

### Pollen dispersal model

Pollen shed and dispersed from the donor anther is the key mediator of gene flow. Here, the pollen donor was acted as the pollen source. The LS model was used to trace a certain amount of pollen grains shed from the donor anther, transported in the air, and finally deposited to the receptor. Then, the distribution of these pollen grains in space and time could be computed from their trajectories.

The direction of the horizontal coordinate axis was determined according to the observed wind vector. The x-axis direction indicated the horizontal wind direction, the y-axis indicated the cross-wind direction, and the z-axis was perpendicular to the ground. All trajectories were modelled independently each other. The movement of pollen grains was decided by the wind velocity^32^:

1 $$\Delta {X}_{i}={U}_{i}\Delta t$$

Where Δ*X* was the displacement of a pollen grain; Δ*t* was a certain time step; *i* = 1, 2, and 3, and they represented the x, y, and z-axis directions, respectively; *U*_*i*_ was a three-dimensional wind vector, and *U*_1_, *U*_2_, and *U*_3_ represented wind speeds in *i* directions, respectively, that was u, v, and w. In the vertical direction, due to induction by gravity, the displacement of a pollen grain in space was affected by the settling speed,

2 $$dz=(w-{v}_{S})dt$$

Where *v*_*s*_ was the settling speed of pollen grain in still air.

An instantaneous wind speed, *U*, that could be divided into a mean part, $\bar{U}$, and a stochastic part or turbulent velocity, $U{\prime}$:

3 $${U}_{i}={\bar{U}}_{i}+{U{\prime} }_{i}$$

The mean in the x direction was derived from observation of the eddy covariance system, while the means in the *y* and *z* directions were assumed to be zero. The turbulent velocity at each moment was calculated from their previous turbulent velocity^33,34^,

4 $${U{\prime} }_{i}(t+\varDelta t)={R}_{iL}(\Delta t){U{\prime} }_{i}(t)+{\sigma }_{i}{(1-{R}_{iL}^{2})\Delta t))}^{0.5}d{\zeta }_{i}$$

where, $d\zeta$ was a set of random numbers independent of each other, normally distributed with mean 0 and variance 1; *R*_*L*_ was a Lagrangian autocorrelation function, which was exponentially related to the Lagrangian timescale *T*_*L*_, ${R}_{iL}=\exp (1-\frac{\Delta t}{{T}_{iL}})$.

Once the pollen grain entered into the canopy, we assumed that all particles were absorbed and no reflection existed. Therefore, the pollen concentration was the amount of pollen deposited at the top of canopy:

5 $$D(i,j)=\frac{Q(m,n)}{{N}_{p}S}\mathop{\sum }\limits_{n=1}^{{N}_{p}}{D}_{n}(i,j)$$

where, *N*_*p*_ represented the number of total particles traced by LS model; *D*_n_ was the amount of donor pollen fallen on the (*i*, *j*) grid unit; and *S* was the area of a grid unit. In addition, *Q*(*m*, *n*) was the pollen grains shedding from the donor plant located at (*m*, *n*) and escaping to the air above the canopy. They could travel in the air and gradually deposit on another plant, so called the “actual pollen source”. However, a lot of pollen grains either remained in the donor anther or they were captured by canopy and deposited *in situ* after shedding, the portion of the actual pollen source to the total pollen grains produced in the donor anther is very little. The portion was empirically determined to be 8% in the field experiment. Finally, the amount of pollen deposited to the (*i*, *j*) was accumulated and compared with the measured pollen deposition.

### Determination of meteorological parameters

The meteorological parameters that the model requires were: average wind speed at the pollen displacement height, $\bar{U}$, velocity variances, ${\sigma }_{u}^{2}$, ${\sigma }_{v}^{2}$, and ${\sigma }_{w}^{2}$, and Lagrangian time scale, *T*_*L*_. These statistics were equal in the horizontal direction. The profiles of them in the vertical direction were related to the friction velocity, u_*_, the Monin-Obukhov length, *L*, which could be obtained indirectly by the eddy covariance system.

Above the canopy, a logarithmic distribution was presented in $\bar{U}$ at the vertical direction^35^:

6 $$\bar{U}=\frac{{u}_{\ast }}{\kappa }[\,\mathrm{ln}(\frac{z-d}{{z}_{0}})+\varphi ]$$

where, *z* was the pollen displacement height; *d* and *z*_0_ was zero plane displacement of 0.67H and aerodynamical roughness length of 0.13H^36^, respectively; *φ* was for stability correction, $$\varphi =\{\begin{array}{cc}4.7\frac{z-d}{L} & L > 0\\ -2\,\mathrm{ln}(\frac{1+\alpha }{2})-\,\mathrm{ln}(\frac{1+{\alpha }^{2}}{2})+2{\tan }^{-1})(\alpha )-\frac{\pi }{2} & L < 0\end{array}$$, and $\alpha ={[1-15(z-d)/L]}^{0.25}$.

Under stable and neutral conditions, the velocity variance and the Lagrangian time scale were respectively^37,38^:

7 $$\begin{array}{ccc}{\sigma }_{u} & = & {\sigma }_{v}=2.4{u}_{\ast }\\ {\sigma }_{w} & = & 1.25{u}_{\ast }\\ {T}_{L} & = & \frac{0.5z}{{\sigma }_{w}}(\frac{1}{1+5z/L})\end{array}$$

Under unstable conditions, they were respectively^38^:

8 $$\begin{array}{ccc}{\sigma }_{u} & = & {\sigma }_{v}={u}_{\ast }{\left[4+0.6{\left(\frac{{z}_{PBL}}{-L}\right)}^{2/3}\right]}^{1/2}\\ {\sigma }_{w} & = & 1.25{u}_{\ast }{\left(1-3\frac{z-d}{L}\right)}^{1/3}\\ {T}_{L} & = & \frac{0.5z}{{\sigma }_{w}}{\left(1,-,\frac{6z}{L}\right)}^{0.25}\end{array}$$

where, *z*_*PBL*_ was the height of the planetary boundary layer, which is considered 1000 m under unstable conditions.

### Water content and CTE of pollen grains for rice included in the model

After a pollen grain was shed from the anther, its movement in a fluid experienced two phases. In the initial phase, due to gravity and inertia, the trajectory of particles could not completely follow the turbulent motion. For small particles, the moment was extremely short and could usually be ignored; however, for heavy particles, it would be necessary to account for the CTE. Sawford & Guest^39^ reasoned that the Lagrangian time-scale of heavy particle should be less than that of passive fluid. The following approach could correct the CTE from heavy particles:

9 $${T}_{P}=\frac{{T}_{L}}{\sqrt{1+{(\beta {v}_{S}/{\sigma }_{w})}^{2}}}$$

where, *β* was an empirical coefficient, taken as 1.5^40^, and *v*_*S*_ was estimated by the Stokes law^20^.

10 $${v}_{{\rm{S}}}=\frac{{d}_{{\rm{p}}}^{2}({\rho }_{{\rm{p}}}-{\rho }_{{\rm{a}}})g}{18\nu }$$

Where, *d*_p_ was the pollen diameter, *ρ*_p_ and *ρ*_a_ represented the density of pollen grains and air, respectively, *υ* was the air viscosity coefficient, and *g* was the acceleration of gravity.

*d*_p_ and *ρ*_p_ were the time-varying parameters, which was determined by the dehydration rate of pollen grain,

11 $${\omega }_{p}={\omega }_{p0}\exp (-0.0006\cdot VPD\cdot \tau )$$

Where, *τ* was the time of pollen exposed in the air (min); *ω*_*p*_ referred to the pollen water content after exposed in the air for τ min; *ω*_*p*0_ was the initial pollen water content, the measured result was about 60.0%; VPD was the vapor pressure deficit, hpa.

Under the initial conditions, the measured diameter of pollen grain for rice was *d*_p0_ = 4.2 × 10^−5^ m, and the density was *ρ*_p0_ = 1.23 × 10^6^ g·m^−3^. The pollen weight was decreased due to water loss:

12 $$m=\frac{{\rho }_{{\rm{p0}}}\pi {d}_{p0}^{3}}{6}(1-{\omega }_{p}/100)$$

After completely losing water, the pollen weight was ${m}_{{\rm{dry}}}=\frac{{\rho }_{{\rm{p0}}}\pi {d}_{p0}^{3}}{6}(1-{\omega }_{p0}/100)$. In the dehydration process, the pollen diameter were decreased as:

13 $$d={\left[{{d}_{0}}^{3}-\frac{6{m}_{{\rm{dry}}}}{\pi {\rho }_{{{\rm{H}}}_{2}{\rm{O}}}}(\frac{1}{1-{\omega }_{p0}/100}-\frac{1}{1-{\omega }_{p}/100})\right]}^{1/3}$$

Where, *d* was the diameter of pollen grains, *ρ*_H2O_ was the density of water. The pollen density, *ρ*_p_, could be obtained from the weight and volume of pollen grains, ${\rho }_{p}=\frac{6m}{\pi {d}^{3}}$.

### Field experimental design

The field experiment was conducted from April to November, 2017 at the Gongqing Tuan Farm (104°5′N, 36°3′E), Xinminzhou, Jingkou District, Zhenjiang City, Jiangsu Province. Conventional rice varieties, “Ling Liang You” and “Te Shan Zhan”, were used as the pollen sources. For “Ling Liang You”, a square design and three treatments, 5 × 5 m^2^ (T1), 10 × 10 m^2^ (T2), and 15 × 15 m^2^ (T3) were set. T3, T1, and T2 were arranged in order from east to west and the separation distance between each treatment was more than 40 m to reduce interference across the experiment. For “Te Shan Zhan”, the special occupation experiment (T4) adopted a rectangular design as a pollen source with 310 m length and 130 m width, which was the largest rice pollen source to date. In order to produce a uniform underlying surface, all pollen sources were adjacent to the surrounding rice, and their sowing date was adjusted so that the pollen source bloomed one to two months earlier than the surrounding rice.

### Meteorological observation

During the flowering period, the microclimate in the field was measured using an eddy covariance system. The wind vector was determined using a three-dimensional sonic anemometer (CSAT-3, Campbell Scientific, USA). The tilt correction was done to make the average wind speed in the crosswind and vertical direction zero. Global radiation was measured by LI-200×(LI-COR Inc., USA), air temperature and relative humidity was measured by HMP155A (Vaisala Corporation, Finnish), and wind speed and direction were measured by 010 C and 020 C (Met One Instruments, Inc., USA). These sensors were all installed in the field at the height of 2.0 m from the ground. They were collected and stored by CR3000 Datalogger (Campbell Scientifics, USA). The sampling frequency was 1 Hz, and the average value was stored every 30 min. Weather phenomena were recorded manually.

### Measurement of potential source strength

After the rice florets opened, stamens rapidly elongated, and then the pollen grains were released from the anthers. The maximum amount of pollen grains shed into the air depended on the area of pollen source and the potential source strength per unit area. The latter was the product of the number of effective panicles per unit area (ear·m^−2^, RP), the number of flowering spikelets per panicle (floreits·pikelet^−1^. FS), and the amount of pollen grains from a single spikelet (grain·spikelet^−1^, PS).

### Measurement of pollen deposition

**Figure 4:**
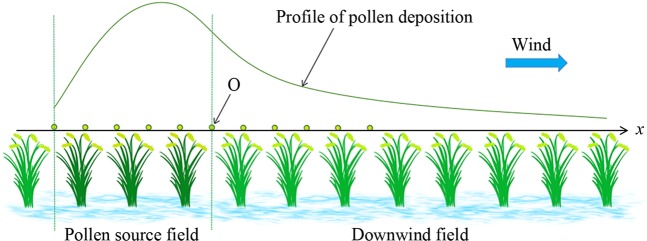
Profile map of the locations for observing the pollen deposition. Note: There are two designs of pollen source. One is a square design with 3 treatments where the source areas are 5 m×5 m (T1), 10 m×10 m (T2), and 15 m × 15 m (T3) respectively; the other is rectangular design with only one treatment, where the length and width of pollen source are 310 m and 130 m (T4), respectively. The black dots are observation points of pollen deposition, which are distributed along the main wind direction from north to south in the central axis of the source area. The observation point “O” is the boundary point between the source area and the downwind area. d is the spacing of adjacent observation points, which are d = 1 m (T1), d = 2 m (T2), d = 3 m (T3), and d = 50 m (T4) in four treatments. “*x*” represents the distance between the observation point and “O”.

The sites for observing the pollen deposition were located on the central axis of the pollen source and were distributed from south to north along the wind direction (Fig. 4). “O” was the boundary point between the pollen source area and the downwind area. Except for “O”, there were five observation points at both the pollen source area and the downwind area in the T1-T3 treatments; the T4 treatment had seven observation points at the pollen source area and one observation point at the downwind area. The spacing distance between the two adjacent observation points were 1 m in T1 treatment, 2 m in T2 treatment, 3 m in T3 treatment, and 50 m in T4 treatment, respectively. “*x*” represents the distance between the location of the observation point and “O”. When *x* is negative, it indicates that the observation point was located in the pollen source area; when *x* is positive, it indicates that the observation point was located in the downwind area of the pollen source.

The experiments used a glass slide coated with petrolatum to capture the pollen grains in the air, and the slide was placed horizontally at the top of the plants. Due to the different plant heights of different varieties, T1-T3 was placed at a height of 80 cm from the ground and T4 was placed at a height of 120 cm from the ground. During the flowering period, the slides were set out at 07:00 daily and retrieved at 16:00 on the same day. The amount of pollen grains in the microscope fields was read and this was repeated 50 times for each slide, and finally the number of pollen grains was added up to calculate the pollen deposition (grain·cm^−2^).

### Simulation design

RP, FS, and PS did not show much difference between the different varieties. Table S1 shows that this difference is not more than 3 times between one variety and another. However, as the area of pollen source increased, the total pollen grains produced in the anther increased geometrically. Thus, the pollen source area was the key factor to the number of the pollen grains released into the air. In this study, we focused on the impact of pollen source area. Here, a total of 18 cases, including six levels of the pollen source area, plus three levels of the wind speed, are designed, which are shown in Table 3 in detail. In order to analyze the relationship between the source area and the pollen deposition, we designed a set of cases with the length and width of pollen source increasing at intervals of 10 m from 10 × 10 m^2^ to 2000 × 2000 m^2^.

**Table 3:** Case design of the pollen dispersal model.

Factor		Level of pollen source area					
		10 × 10 m^2^	20 × 20m^2^	50 × 50 m^2^	100 × 100 m^2^	200 × 200 m^2^	300 × 300 m^2^
Wind speed	0.5 u	Case1	Case2	Case3	Case4	Case5	Case6
	1.0 u	Case7	Case8	Case9	Case10	Case11	Case12
	1.5 u	Case13	Case14	Case15	Case16	Case17	Case18

Note: 0.5 u, u and 1.5 u indicate that the simulated wind speed is 0.5 times, 1 time, and 1.5 times of that in the ‘Ling liang you’ experiment.

## Supplementary information

Supplementary information.
