# Supplementary material for: Increased pollen source area does not always enhance the risk of pollen dispersal and gene flow in Oryza sativa L
Source: Sci Rep. 2020 Apr 9;10:6143. doi: 10.1038/s41598-020-63119-z (PMC7145849; doi:10.1038/s41598-020-63119-z)
Supplement: Supplementary file 1 — Supplementary information. [file 41598_2020_63119_MOESM1_ESM.pdf]

Increased pollen source area does not always enhance the risk of pollen dispersal and gene flow in *Oryza sativa* L.

Ning Hu[1,2], Xiaodong Jiang[2], Qianhua Yuan[3], Wuge Liu[4], Kemin Yao[2], Yan Long[5], Xinwu Pei[5]

1 Yale-NUIST Center on Atmospheric Environment, International Joint Laboratory on Climate and Environment Change, Nanjing University of Information Science & Technology, Nanjing 210044, China
2 Jiangsu Key Laboratory of Agriculture Meteorology, School of Applied Meteorology, Nanjing University of Information Science and Technology, Nanjing, 210044, China
3 College of Tropical Agriculture, Hainan University, Haikou 570228, China
4 Rice Research Institute, Guangdong Academy of Agricultural Sciences, Guangzhou, 510640, China
5 Biotechnology Research Institute, Chinese Academy of Agricultural Sciences, Beijing 100081, China

Table S1. Comparison of the number of effective panicles per unit area (RP), the number of flowering spikelets per panicle (FS), the amount of pollen per plant (PG), and the pollen source strength per unit area (PS) between different varieties.

Item	Wuyunjeng	Jifeng B	Zhongjiu A	Teshanzhan	Lingliangyou
RP (panicle m^{-2})	305.9±74.1 b	378.4±60.4 a	345.1±75.2 ab	207.8±54.1 c	121.6±31.8 d
FS (flower $panicle^{-1}$)	93.0±34.0 b	86.3±52.3 b	51.4±28.0 c	141.7±35.9 a	107.6±9.2 ab
PG (grain $flower^{-1}$)	1040±438 d	2020±553 a	1600±562 bc	1860±615 ab	1530±710 c
PS (grain m^{-2})	2.96×10^7	6.60×10^7	2.84×10^7	5.48×10^7	2.00×10^7

Note: Wuyunjeng, Jifeng B, Zhongjiu A, Teshanzhan, and Lingliangyou are the name of rice varieties.

Lower case letters indicate a Duncan test at the 0.05 significance level. The same letter indicates no significant difference between different varieties.
